# Changes in body composition and phase angle from diagnosis to gastrostomy, in motor neuron disease patients: a longitudinal study

**DOI:** 10.1038/s41430-026-01702-9

**Published:** 2026-02-07

**Authors:** Jie Yang, Yun Zhao, Mario Soares, Merrilee Needham, Andrea Begley, Emily Calton

**Affiliations:** 1 Department of Nutrition and Dietetics, Fiona Stanley Hospital, Perth, WA Australia; 2 Curtin School of Population Health, Faculty of Health Science, Curtin University, Perth, WA Australia; 3 Division of Nutrition, St John’s Research Institute, St. John’s National Academy of Health Sciences, Bangalore, Karnataka India; 4 Department of Neurology, Fiona Stanley Hospital, Perth, WA Australia; 5 School of Medicine, The University of Notre Dame Australia, Perth, WA Australia; 6 Centre for Molecular Medicine & Innovative Therapeutics, Murdoch University, Perth, WA Australia; 7 Perron Institute, QEII Medical Centre, Perth, WA Australia; 8 Curtin School of Allied Health, Faculty of Health Science, Curtin University, Perth, WA Australia; 9 Fiona Stanley Fremantle Hospitals Group, Perth, WA Australia

**Keywords:** Nutrition, Neurodegenerative diseases

## Abstract

Limited studies have explored the longitudinal alterations of fat-free mass (FFM), fat mass (FM), and phase angle (PhA) in the motor neuron disease (MND) population. This pilot longitudinal study investigated body composition changes via bioimpedance analysis (BIA) at time of diagnosis and gastrostomy. Nineteen patients (*n* = 11, 57.9% females) were included. Statistically significant reductions in FFM [median (IQR) 43.0 (17.8) kg to 42.1 (16.7) kg, *p* < 0.001] and PhA [4.6 (2.0)° to 4.5 (2.1)°, *p* = 0.012] were found. FFM (*p* = 0.007, 95% CI 0.505, 1.32) and FM (*p* = 0.024, 95% CI 0.160, 1.249) were significant predictors of weight change, with FFM accounting for 72% of the change. Larger longitudinal studies are needed to explore the clinical benefit associated with reducing pre-gastrostomy FFM loss in the MND population. In the interim, clinicians should consider monitoring body composition and implementing interventions aimed at preserving body weight and composition, particularly FFM, prior to gastrostomy placement.

## Introduction

Motor neuron disease (MND) is a progressive neurodegenerative disorder with a 3–4 year average survival from symptom onset [1]. Gastrostomy feeding is commonly used to support patients who develop severe dysphagia [2]. Prior research demonstrates that weight loss in the interval between diagnosis of the condition and gastrostomy as a major predictor of mortality on day 30, 90 and 180 post-gastrostomy [3]. While differences in body composition exist between MND and healthy people [4, 5], there is a lack of research investing the longitudinal changes in body composition during the progression of MND.

Therefore, our primary aim was to understand the magnitude of the change in fat-free mass (FFM) and fat mass (FM) in MND patients in the period from diagnosis to gastrostomy. We used bioelectrical impedance analysis (BIA), a widely used technique for body composition measurement that is a reliable method for assessing nutritional status in clinical settings, including the MND population [4, 5].

## Methodology

This pilot retrospective longitudinal study included MND patients with a gastrostomy treated at a tertiary hospital in Western Australia between 01 January 2015 and 31 December 2021. Full details of the methodology have been previously published [6].

Age, sex, weight and height were collected at the time of diagnosis and again on admission for gastrostomy insertion. Patients were free living prior to admission and the median (interquartile range [IQR]:) duration between diagnosis and gastrostomy insertion was 239 [217] days. Body composition from BIA (Bodystat multiscan 5000, Bodystat Ltd, Douglas, Isle of Man, British Isles) was also measured at these two timepoints, with the participant in the supine position. The 50 kHZ frequency was used, as per Desport and colleagues [7, 8]. FM (kg and %), FFM (kg and %) were estimated from manufacturer’s equations, while phase angle (PhA) was calculated by the formula: PhA = arctan (Xc/R) × (180/π), where Xc is the body reactance and R the bioelectrical resistance. BMI was calculated using the formular weight (kg)/ height (m^2^).

Descriptive statistics were used to summarise the characteristics of the study population. Frequencies and proportions were used for categorical data and median (IQR) for continuous data. To compare differences in body composition at the time of diagnosis and gastrostomy, Wilcoxon signed-rank was used for all data due to the small sample size. Multiple linear regression (bootstrap method) analysis was used to identify the predictors of weight change from diagnosis to gastrostomy insertion. Given the small sample size of BIA data, we only included variables well-known to influence body composition: age, sex, FFM and FM. Statistical significance was set at *p* < 0.05. SPSS*®* (Statistical Product and Service Solutions, Windows Version 27, IBM, Australia) was used for statistical analysis.

## Results

The study cohort comprised 19 patients, 11 (57.9%) females and 8 (42.1%) males and 16 participants (84.2%) had bulbar onset. The median (IQR) age was 65 (14) years at diagnosis and gastrostomy insertion. The median (IQR) interval between BIA measurements was 239 (217) days.

The change weight, BMI, body composition and phase angle between diagnosis to gastrostomy is shown in Table 1. Overall, there was a statistically significant decrease in weight kg (*p* = 0.002), BMI kg/m^2^ (*p* = 0.002), FFM kg, (*p* < 0.001), and PhA ° (*p* = 0.012) from diagnosis to gastrostomy. When examining FM and FFM as the % of body weight, no significant statistical differences were observed over time. However, there was a noticeable decrease in FFM percentage with a concurrent increase in FM percentage.

**Table 1:** Changes in weight, body composition and phase angle between diagnosis and gastrostomy insertion (*n* = 19).

Variable	Diagnosis	Gastrostomy	*P* value
	median (IQR)	median (IQR)	
Weight (kg)	68.4 (25.5)	64.0 (20.0)	**0.002**
BMI (kg/m^2^)	23.7 (7.18)	21.9 (6.4)	**0.002**
FFM (kg)	43.0 (17.8)	42.1 (16.7)	**<0.001**
FM (kg)	18.6 (9.6)	18.1 (9.2)	0.807
FFM (%)	69.3 (14.5)	66.6 (12.8)	0.142
FM (%)	30.7 (14.5)	33.4 (12.8)	0.125
PhA (°)	4.6 (2.0)	4.5 (2.1)	**0.012**

*P* value: change in body composition from diagnosis to gastrostomy was assessed by Wilcoxon signed-rank test.

Bold denotes statistical significance (p < 0.05).

*BMI* Body mass index, *FFM* fat-free mass, *FM* fat mass, *IQR* interquartile range, *PhA* phase angle.

Multiple linear regression (adjusted r square = 0.880), showed that change in FFM kg (*p* = 0.007, 95% CI 0.505, 1.329) and FM kg (*p* = 0.024, 95% CI 0.160, 1.249) significantly predicted weight change kg. FFM predicted 72% and FM 28% of the weight change. Age at gastrostomy (*p* = 0.365, 95% CI −0.115, 0.229) and sex (male) (*p* = 0.287, 95% CI −5.888, 1.285) were not significant predictors of weight change.

## Discussion

Our findings indicated that FFM loss, along with PhA reduction was the predominant changes during the weight loss experienced by MND patients. These results were similar to a large study with 74 MND patients [5], which reported statistically significant losses in weight, FFM and PhA from diagnosis to pre-death. Although the increase we observed was not statistically significant, FM percentage increased from 30.7% to 33.4%, in line with previous literature [5].

Several possible hypotheses have been proposed to explain the loss of FFM and relatively preserved FM in MND. Firstly, neurodegeneration of motor neurons accelerate muscle loss [5]. Secondly, FFM loss occurs due to reduced caloric intake from dysphagia and loss of appetite [9]. As typical caloric restriction leads to change of both FFM and FM stores, the selective depletion of FFM suggests that metabolic processes in MND may preferentially target lean tissue, pointing to altered energy metabolism beyond simple undernutrition. Lastly, MND induces a hypermetabolic state [10], hypothesised to stem from increased respiratory muscle expenditure [10]. Future research should investigate whether neuromuscular changes drive shifts in substrate utilisation and metabolic flexibility with nutritional intake, to better inform targeted nutritional strategies.

While not all FFM loss is modifiable, some may be and this deserves clinical attention. In light of the inevitable denervation-induced skeletal muscle atrophy in MND, a delay in nutritional intervention may exacerbate the loss of muscle mass. As a pragmatic diagnostic approach to detecting malnutrition in MND patients, it has been previously advised to monitor BMI and employ body composition assessment concurrently [8]. Our observations support the rationale for considering interventions aimed at preserving body weight and composition, particularly FFM, prior to gastrostomy placement.

We acknowledge our study has some limitations. Our study involved a small sample size. Further, the interval between BIA measurements varied among participants. Future research should be directed towards identifying exercise and nutrition interventions that minimise FFM losses in the context of MND, prior to gastrostomy placement, to enhance its utility in clinical practice.

In conclusion, our study provided longitudinal data on the changes in the body composition and phase angle of MND patients from diagnosis to gastrostomy insertion and identified that weight loss is largely characterised by FFM loss. Clinicians are recommended to monitor body composition and future research should explore interventions aimed at preserving body weight and composition, particularly FFM, prior to gastrostomy placement.

## Data Availability

The data that support the findings of this study are available from the corresponding author upon reasonable request.
